# Terahertz sensing of reduced graphene oxide nanosheets using sub-wavelength dipole cavities

**DOI:** 10.1038/s41598-023-39498-4

**Published:** 2023-07-31

**Authors:** Vaishnavi Sajeev, Shreeya Rane, Debal Ghosh, Nityananda Acharyya, Palash Roy Choudhury, Arnab Mukherjee, Dibakar Roy Chowdhury

**Affiliations:** 1 Ecole Centrale School of Engineering (ECSoE), Mahindra University, Hyderabad, 500043 India; 2 Central Glass and Ceramic Research Institute (CGCRI), Kolkata, 700032 India

**Keywords:** Materials science, Nanoscience and technology, Optics and photonics, Physics

## Abstract

Because of extraordinary optoelectronic properties, two-dimensional (2D) materials are the subject of intense study in recent times. Hence, we investigate sub-wavelength dipole cavities (hole array) as a sensing platform for the detection of 2D reduced graphene oxide (r-GO) using terahertz time-domain spectroscopy (THz-TDS). The r-GO is obtained by reducing graphene oxide (GO) via Hummer's method. Its structural characteristics are verified using X-ray diffraction (XRD) and Raman spectroscopy. We also assessed the morphology and chemistry of r-GO nanosheets by scanning electron microscopy (SEM), energy-dispersive X-ray spectroscopy (EDAX), and Fourier Transformed Infrared (FTIR) spectroscopy. Further, we studied the surface plasmon resonance (SPR) characteristics of r-GO nanosheets hybridized dipole cavities using THz-TDS by varying the r-GO thickness on top of the dipole cavities, since these cavities are well known for sustaining strong SPRs. Based on these, we experimentally obtained a sensitivity of 12 GHz/µm for the porous r-GO film. Thus, a modification in SPR characteristics can be employed towards the identification and quantification of r-GO by suitably embedding it on an array of dipole cavities. Moreover, we have adopted a generic approach that can be expanded to sense other 2D materials like Boron Nitride (BN), phosphorene, MoS_2_, etc., leading to the development of novel THz nanophotonic sensing devices.

## Introduction

Two-dimensional (2D) materials have emerged as a new class of materials with unique and extraordinary properties that have the potential to revolutionize various fields of science and technology. They possess excellent optical, electronic, chemical, and mechanical properties due to their ultra-thin and planar geometry^1^. Among all the 2D materials studied till now, graphene, a two-dimensional layer of carbon atoms arranged in a honeycomb lattice, is the most well-known and extensively studied material^2^. Another interesting 2D carbon material is graphene oxide (GO) which is a graphene derivative that has attracted a lot of interest because of its tunable properties, ease of synthesis, and ability to form thin films^3,4^. In addition, reduced graphene oxide (r-GO), which is derived from GO by removing oxygen functional groups, has a lower oxygen content and higher electrical conductivity and has received a lot of attention in recent years because of its potential applications in fields like electronic devices, energy storage, biosensors, and biomedical devices^5–8^. Furthermore, r-GO has more chemical and thermal stability than GO due to the removal of oxygen-containing functional groups, making it appropriate for applications in severe chemical and high-temperature conditions^9–11^. Besides the higher conductivity and stability shown by r-GO, the presence of chemically active defect sites^12^ makes it an excellent candidate for various sensing applications, including biosensors^13^. Moreover, it has also exhibited excellent photothermal response due to its strong near-infrared absorption^14^. When exposed to near-infrared light, r-GO produces heat that selectively destroys cancer cells while leaving healthy cells intact, making it a potential candidate for photothermal therapy, a treatment method that uses light to generate heat and destroy cancer cells. In addition to its potential as a cancer therapy, r-GO is used in drug delivery systems due to its vast surface area and ability to interact with medicines through π-π stacking and electrostatic interactions^15^. The biocompatibility of r-GO has also been revealed by various in vitro and in vivo studies^16^. Despite these aforementioned advantages, r-GO is toxic to certain cell types and organisms, raising concerns about its safety for use in biological applications. Recent studies have revealed that r-GO may induce tissue damage and inflammation in animals as well as oxidative stress and DNA damage in cells^17^. Given these potential dangers, it is critical to build sensing devices capable of detecting the presence of r-GO in a variety of situations. It can be able to limit the hazards connected with r-GO use and assure its safe applications in diverse situations by detecting its presence in real time. Therefore, in this work, we explore non-contact r-GO detection utilizing suitable plasmonic devices in the THz domain.

Dipole cavities (hole arrays), which are plasmonic artificial structures (basically, metamaterials^18–20^) made up of an array of sub-wavelength holes, have shown great potential as a sensing platform^21,22^. These cavities are constructed from a regular grid of subwavelength-sized holes that are arranged in a periodic manner. The extraordinary transmission (EOT), which defies the conventional Bethe's aperture theory, is one of the most remarkable optical phenomena found in the array of dipole cavities^23^. This phenomenon is caused by surface plasmon resonance (SPR) and involves increased light transmission through the holes in comparison to the hole area and thus the name extraordinary transmission. This effect occurs when light interacts with an array of dipole cavities, causing surface plasmon polaritons (SPP) to be excited and propagate along the metal–dielectric interface. As a result, the electromagnetic field surrounding the holes is considerably amplified, resulting in improved light transmission through the dipole cavities^24,25^. Depending on the size, shape, inclusion, and arrangement of the cavities, dipole cavities display a variety of novel optical properties, such as negative refractive index, increased transmission or reflection, and polarization-dependent behavior^26–29^. In comparison to other sensing platforms such as split-ring resonators, fishnet structures, or photonic crystals, dipole cavities have several advantages such as relatively simpler fabrication processes (typically one step fabrication), compatibility with a wide range of substrates, and precise control over the optical properties of the material^30–33^. Dipole cavity-based analyte sensing techniques have also invited considerable attentions in recent times^34^. The sensor responses can be designed to suit specific analytes by configuring the electromagnetic properties of the dipole cavity making it versatile. The analytes interact with the electromagnetic field close to the cavity when a sample is introduced in a dipole cavity altering the optical properties of the array. This change can be detected by changes in the EOT or SPR signals, allowing the analyte to be detected with higher sensitivity^35,36^. In addition, dipole cavities have been thoroughly studied for their optical and electromagnetic properties, including in the terahertz (THz) frequency ranging between 0.1 and 10 THz (or wavelengths between 30 to 3,000 µm)^37,38^. The modulation of SPPs at THz has been shown using several THz sub-wavelength plasmonic structures. For instance, SPPs at THz frequencies can propagate over longer distances compared to higher frequencies and can interact effectively with intramolecular and intermolecular vibrations in molecular and biomolecular systems^39,40^. Remarkably, dipole cavities can define effective permittivity, which enables them to sustain SPR modes even at lower frequencies^41^.

Based on these backgrounds, here, we explore the potency of dipole cavities for r-GO sensing employing THz time-domain spectroscopy (THz–TDS). The successful reduction of GO to r-GO using the Hummer’s method is confirmed using X-ray diffraction (XRD), Raman spectroscopy and FTIR spectroscopy while the morphology and elemental composition of the r-GO film is characterized via scanning electron microscopy (SEM) and energy-dispersive X-ray spectroscopy (EDAX). By utilizing THz-TDS, we observed a shift in the SPR frequency in response to changes in the thickness of the r-GO layer on top of the dipole cavities. This shift in the SPR frequency allows for the quantification of r-GO thickness on the surface of the dipole cavities. Subsequently, we experimentally and theoretically calculated the sensitivity of the dipole cavities for r-GO sensing forming a basis for detection of other 2D materials.

## Sample preparation and fabrication

Reduced graphene oxide particles are synthesized by Hummer’s method which involves the oxidation of graphite powder to form graphite oxide, followed by exfoliation to obtain graphene oxide^42^. For this, the following chemicals are used as received: graphite powder (< 20 µm, synthetic), sodium nitrate, ascorbic acid, concentrated sulfuric acid, 30% hydrogen peroxide, concentrated hydrochloric acid, and potassium permanganate (Merck). Deionized Water (18 MU) used for hydrolysis is obtained from a Milli-Q System (Millipore).

This method uses excess Sulphuric acid (H_2_SO_4_) as the solvent, along with Sodium Nitrate (NaNO_3_) and Graphite powder as as an oxidizing agent and the precursor material respectively. To prevent the temperature from increasing above 20 ºC, the mixture is cooled in an ice bath, avoiding the formation of unwanted byproducts. To initiate the oxidation of graphite powder, Potassium Permanganate (KMnO_4_) is slowly added to the mixture. To ensure that the graphite is completely oxidized, the solution is diluted with 5% hydrogen peroxide (H_2_O_2_) solution, which causes the solution to turn brownish-yellow. This solution is then stirred to promote the exfoliation of the graphite oxide which is collected by filtration. The obtained GO is then washed with 1N Hydrochloric acid (HCl) and de-ionized water to remove any remaining impurities. To obtain r-GO, the GO powder is mixed with Ascorbic acid (vitamin C) as a reducing agent and sonicated in an aqueous medium. The obtained r-GO solution is collected by filtration and dried under vacuum at room temperature to obtain r-GO powder. Further, to prepare the r-GO films, we employed the drop-casting method^43^. For that, 0.024 g of r-GO powder is added to 5 ml of ethanol, followed by ultrasonication to ensure uniform distribution. This r-GO solution is drop-casted on the high resistance intrinsic Silicon substrate (> 5,000Ω-cm) and subsequently dried at room temperature for 3 h. The thickness of the r-GO layer for different samples is controlled by carefully varying the number of drops.

We employed a standard UV photolithography, on a silicon substrate of thickness 400 µm in a cleanroom environment to fabricate the dipole cavity samples. The intrinsic silicon wafer is coated with a layer of positive photoresist utilizing spin-coating (at 5,000 rpm). Before being exposed to UV light, a chrome mask is utilized. Subsequently, the photoresist is developed to remove the exposed areas and leave behind the patterned photoresist. Then, using an e-beam deposition process, an Aluminum (Al) layer of 200 nm thick is deposited on the patterned substrate. The residual photoresists are then eliminated using a lift-off procedure, leaving behind a hole array pattern as seen in Fig. 3a (optical micrograph). The r-GO is deposited onto the array of dipole cavities by the drop-casting method, followed by evaporation of the solvent under ambient conditions, which ultimately leads to the formation of thin r-GO film on top of the dipole cavities.

## Characterization

**Figure 1:**
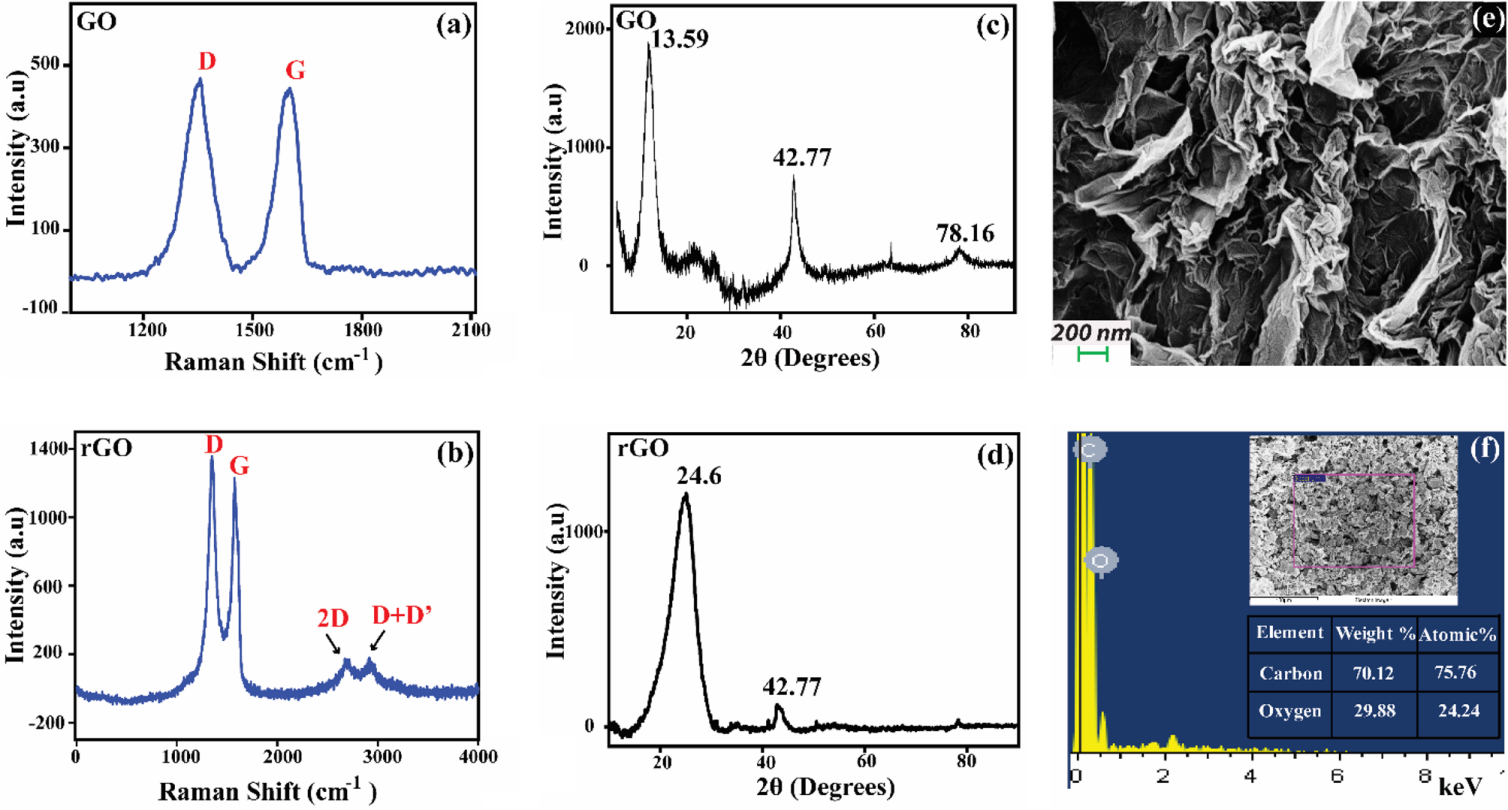
Material characterizations of GO and r-GO: Raman spectra of GO (**a**) and r-GO (**b**) showing characteristic peaks. XRD of GO (**c**) and r-GO (**d**). SEM image of r-GO showing the nanosheet-like structure and (**e**) EDAX spectrum of r-GO along with the weight and atomic percentages of carbon and oxygen in the sample (**f**).

In this study, we have characterized GO and r-GO using Raman spectroscopy (Fig. 1a,b) and XRD (Fig. 1c,d). The XRD data show 2θ peaks at 13.59°, 42.77°, and 78.16° as indicated in Fig. 1c ^44^. These peaks correspond to the (001), (100), and (110) crystallographic planes of GO with interlaminar spacings of 0.65 nm, 0.22 nm, and 0.15 nm respectively. On the other hand, r-GO exhibited peaks at 24.60° and 42.77° (Fig. 1d), which are attributed to the (002) and (100) crystallographic planes, with spacings of 0.36 nm and 0.22 nm respectively^45^. These results confirm the successful reduction of GO to r-GO and the presence of graphene-like domains in the r-GO sample. Further, we performed Raman spectroscopy to confirm the phase and purity of GO and r-GO. The Raman spectra for GO show two prominent peaks, the D and G peaks, located at 1350 cm^−1^ and 1600 cm^−1^, respectively^46^. The D peak arises due to the presence of defects and disorder in the sp^2^ carbon lattice of GO, while the G peak corresponds to the in-plane vibration of the carbon–carbon (C–C) bond from sp^2^ hybridized orbitals^47^. For the r-GO samples, the Raman spectra show additional peaks compared to the GO samples, including the D, G, 2D, and D + D' peaks^46^. The D and G peaks are observed at similar positions to those of the GO sample, while the 2D peak is located at ~ 2700 cm^−1^ and the D + D' peak is located at ~ 2920 cm^−1^. The D + D' peak is a combination of the D and D' peaks, whereas the 2D peak is associated with the two-phonon resonance process. Moreover, the reduction from GO to r-GO has been further verified using the FTIR measurement which is shown in Fig S1 (please refer to the supplementary information). Furthermore, the SEM images of both GO and r-GO indicate a nanosheet like structure and a rough surface^48^ as shown in Fig. S2 and Fig. 1e respectively. The higher thickness of the r-GO material implies that the individual layers of r-GO are stacked on top of each other, resulting in a three-dimensional structure rather than a visibly distinct two-dimensional sheet. The particles are somewhat irregular in shape, with some wrinkles and folds indicating considerable flexibility and a vast surface area. In addition to this, Energy Dispersive X-Ray (EDAX) analysis of GO (Fig. S3) and r-GO (Fig. 1f) reveals the presence of carbon and oxygen atoms, with carbon being the dominant element^49^. From the EDAX analysis, the carbon weight percentage in r-GO is found to be about 70.1%, and the oxygen weight percentage is about 29.9%. Furthermore, the atomic proportion of carbon is around 75.8%, whereas the atomic percentage of oxygen is approximately 24.2%.

**Figure 2:**
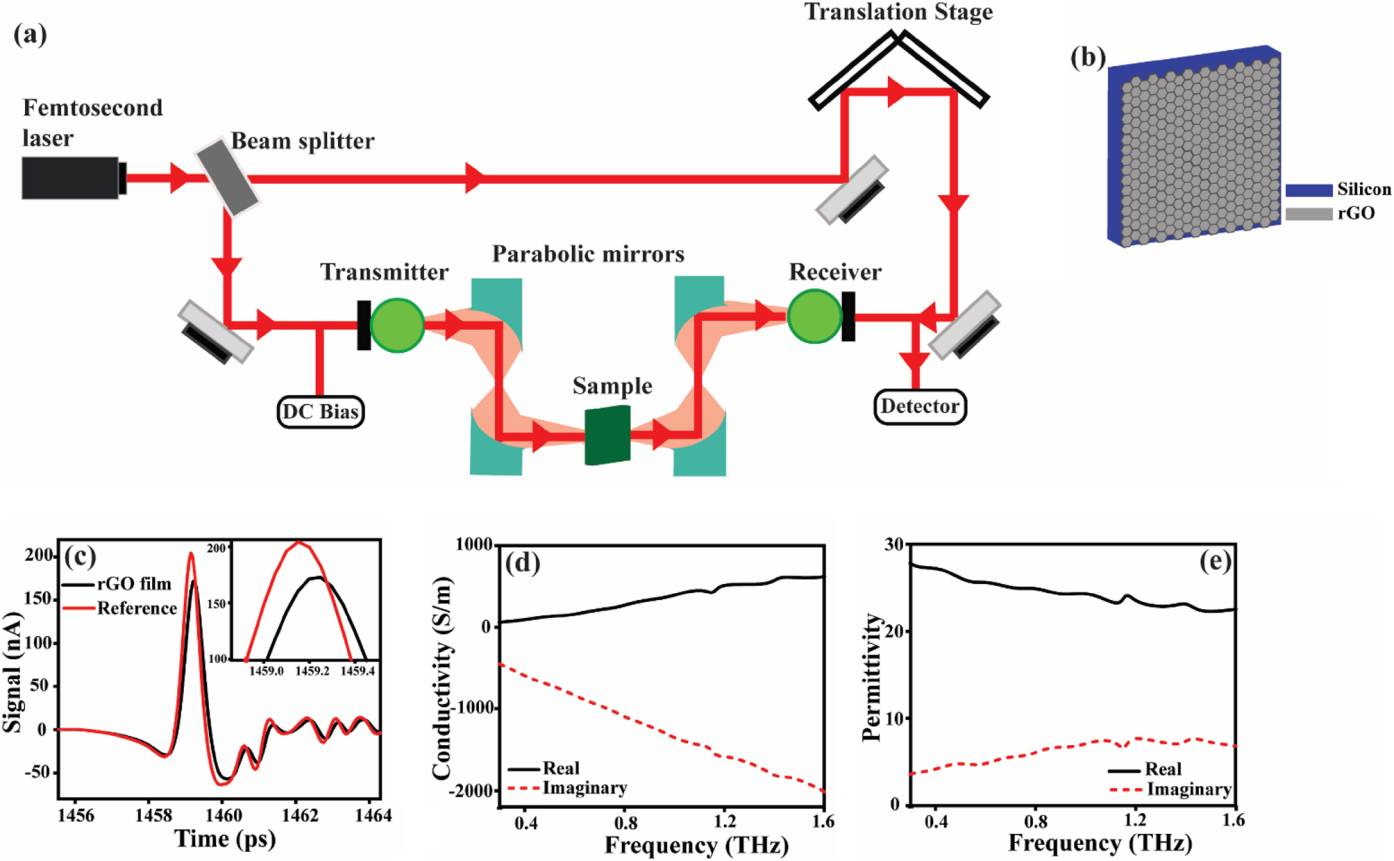
THz characterization of r-GO film (**a**) Experimental setup of THz–TDS for measuring the THz characteristics of r-GO film (**b**) Schematic representation of the r-GO sample on top of the Si substrate. Blue and grey colors represent the Si and r-GO film respectively. (**c**) Time-domain pulse profile of the r-GO film (black) along with the reference (red) acquired through THz TDS. (**d**) Frequency-dependent conductivity of the r-GO film, showing the real (black solid line) and imaginary (red dotted lines) parts of conductivity. (**e**) Frequency-dependent real and imaginary parts of permittivity of the r-GO film, shown by black solid and red dotted lines, respectively.

We have conducted THz transmission studies through the r-GO films to capture the SPR characteristics in the frequency domain, as shown in Fig. 2a. The experiment is conducted in a dry environment using Toptica Teraflash THz–TDS setup to characterize the transmission properties of our samples^50^. The schematic of the r-GO film used in our study is shown in Fig. 2b. We used a pulsed THz generator and a sensitive detector to study the THz time-domain signals. THz TDS measurements are carried out by guiding the THz beam through the sample using two pairs of off-axis parabolic mirrors. The THz-TDS setup comprises a pair of photoconductive antennas (PCAs) designed on InGaAs/InP substrates to generate THz pulses, as well as a receiver to detect the transmitted pulses through the samples^51^. The THz radiation is generated using a femtosecond laser (wavelength of 1560 nm) with an approximate repetition rate of 100 MHz and a pulse width of < 60 fs. The incoming THz pulse is modulated as it travels through the sample, and the laser pulse probes this modulation effect at the detector side. The detector uses the pump–probe technique to evaluate the transmitted THz pulse through the samples in the temporal domain^52,53^. Laser pulse at the detector side pumps photocarriers and THz field probes the carriers in order to provide information about the generated THz pulse. Figure 2c shows the time-domain pulse profile of the r-GO film (black) along with the reference pulse (red). The pulse profile shows considerable changes, demonstrating that the r-GO coating has a major impact on THz radiation transmission. The pulse profile of the r-GO film shows a reduction in amplitude and a shift in time delay compared to the reference pulse. A bare silicon substrate of the same size and dimensions as the r-GO deposited samples are examined under similar experimental conditions to account for any external effects that may affect the THz transmission. This is used as a reference measurement to normalize the THz signals transmitted through the r-GO samples. The contribution from any system components or the environmental conditions unrelated to the samples is eliminated by normalizing the data obtained using the reference, allowing for a reliable study of the transmission properties of the samples. The normalization also adjusted for any system drift that may have happened, assuring consistent and reliable results. The time-domain response of the transmitted THz signal is converted to the frequency domain using Fast Fourier transformation (FFT). Further, we extracted conductivity and permittivity using the Lorentz-Drude model which is found to be frequency-dependent, with the real and imaginary parts exhibiting different behavior in the THz frequency range as shown in Fig. 2d,e respectively. Similarly, the transmission spectra are recorded for the array of dipole cavities for three different configurations: without r-GO, with a 4 µm thick r-GO, and with an 8 µm thick r-GO, followed by FFT. We ensured that the characterization is performed with utmost care and precision to obtain accurate and reliable results for the analysis of the dipole cavity samples.

## Numerical simulations

**Figure 3:**
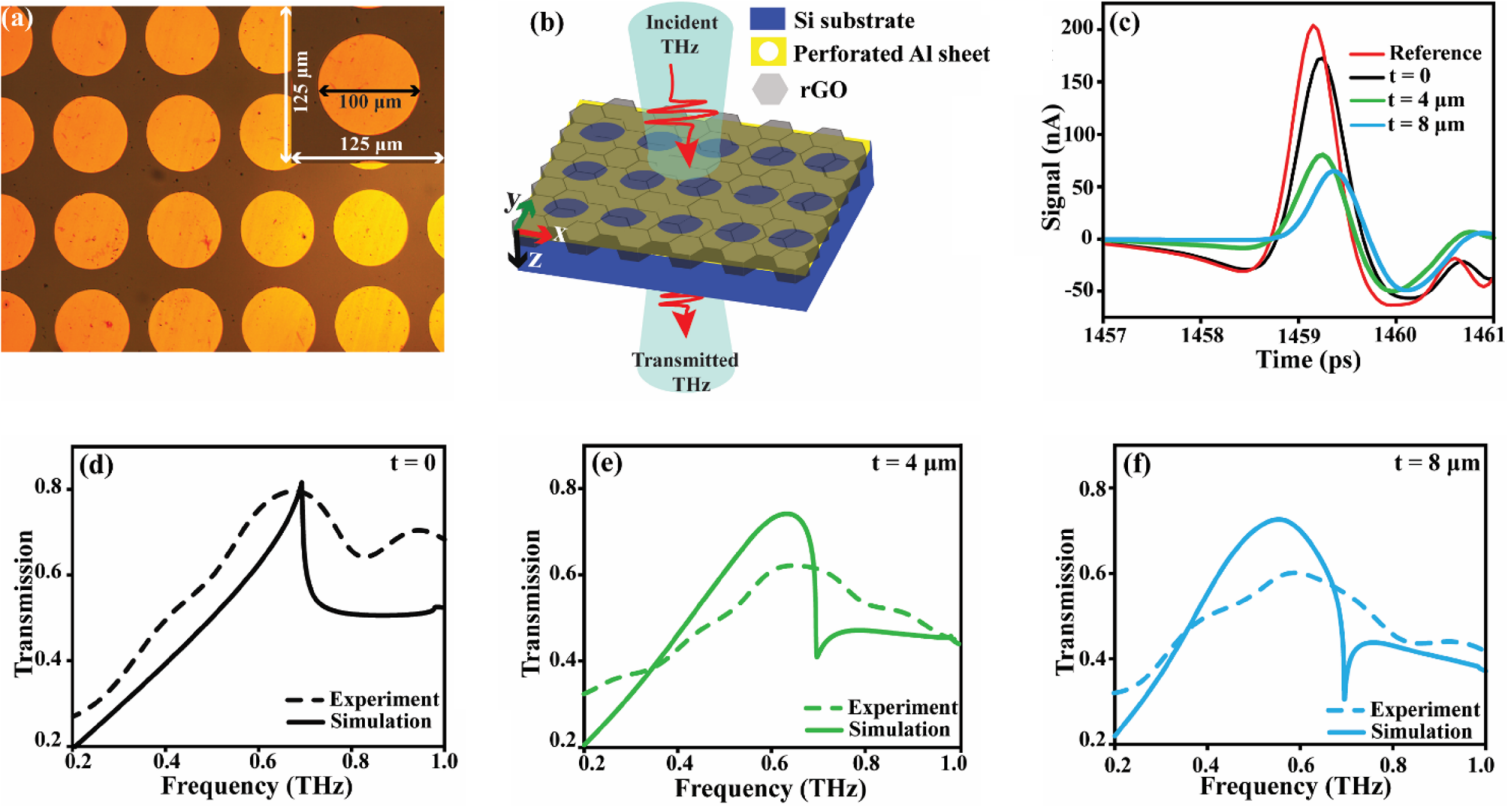
(**a**) Optical micrograph of the fabricated array of dipole cavities on top of the silicon substrate depicts the regular and uniform distribution of holes with a diameter of 100 μm and a periodicity of 125 μm. (**b**) Schematic representation of THz wave propagation through the array of dipole cavity patterned on a silicon substrate (dark blue) with a 200 nm Al layer (yellow) and a hexagonal-shaped r-GO layer (grey) on top of it. The incident THz wave (shown in light blue) is transmitted through the sample. (**c**) Time domain pulses of the reference (red), dipole cavities without r-GO (black), dipole cavities with r-GO of thickness t = 4 µm (green), and r-GO with thickness t = 8 µm (cyan). (**d**–**f**) show the THz transmission spectra of the array of dipole cavities without r-GO, with r-GO of thickness t = 4 µm, and t = 8 µm respectively. The solid lines represent the simulation data, while the dashed lines correspond to the experimental results.

We have used the commercial software CST Microwave Studio to perform electromagnetic simulations of the dipole cavities with and without r-GO. CST solves Maxwell's equations in the frequency domain using the finite integral technique (FIT). In this approach, the region of interest is discretized into small-volume elements. The electric and magnetic fields at the center of each element are calculated using the curl and divergence equations. A 2D square lattice of periodicity (P) 125 μm is modeled on a silicon substrate of 400 μm thickness and with a permittivity of 11.9. The permittivity of r-GO is extracted from experimental measurements and loaded into the CST simulation to model the inclusion of r-GO. The simulations are pursued using a unit cell boundary condition along both the positive and negative x- and y- axes, and an open, open add space along the negative and positive z-axes. Here, the unit cell comprised of a circular dipole cavity with a hole width of 100 μm, arranged in a square lattice with a periodicity of 125 μm (Fig. 3a). The unit cell dimensions for the simulation are chosen to be the same as the fabricated samples. The simulation uses a tetrahedral meshing with an approximate grid size of 12,000 tetrahedrons to generate the array for the fast and accurate calculations.

## Results and discussion

We have carried out THz–TDS (see Fig. 3b) by varying the thicknesses of r-GO (t = 0, t = 4 μm, t = 8 μm) to examine the thickness dependency of r-GO on the transmission characteristics of dipole cavities. The THz–TDS studies revealed a significant resonant response in the dipole cavities transmission spectra, which is attributed to the SPP excitation at the metal–dielectric interface. The EOT phenomenon occurs when a periodic array of subwavelength apertures or cavities in a metal sheet over a dielectric substrate allow more light to pass through than would be anticipated based on the size of the individual apertures^25^. EOT is a consequence of both the diffractive coupling of the incident light to the SPPs and the excitation of SPPs on the metal–dielectric interface, which results in high-field confinement and localization.

In addition, momentum conservation and phase matching must be satisfied for the SPPs and diffracted light to couple efficiently. Momentum conservation requires that the wavevector of the incident light be equal to the sum of the wavevector of the SPP and the diffracted wavevector of the grating, while phase matching ensures that the SPP and the diffracted wave are in phase, leading to constructive interference and enhanced transmission^54,55^. The transmission maxima in the spectra of the diffracted wave from a metal surface are closely related to the periodicity of the surface corrugation and the corresponding material properties of the interfacial media^56^. The SPR peak and Wood's anomaly (WA) which is the transmission minimum for normal incidence can be described by the following equations:

1 $$\lambda_{spp} \left( {i,j} \right) = \frac{P}{{\sqrt {i^{2} + j^{2} } }}\sqrt {\frac{{\varepsilon_{1} \varepsilon_{2} }}{{\varepsilon_{1} + \varepsilon_{2} }}}$$

2 $$\lambda_{WA} \left( {i,j} \right) = \frac{P}{{\sqrt {i^{2} + j^{2} } }}\sqrt {{ }\varepsilon_{2} { }}$$

In Eqs. (1) and (2) for the SPR peak and WA, i and j represent the integer corresponding to the modes, whereas ε_1_ is the permittivity of the metal and ε_2_ is the permittivity of the Silicon. At a periodicity of 125 µm, our analysis using Eq. 1 indicates that the array of dipole cavities sustains surface plasmon modes corresponding to (1,0). The SPR exhibited a shift towards lower frequencies with increasing thickness of r-GO, as shown in Fig. 3d–f. The SPR is observed at a frequency of 0.69 THz in the absence of r-GO, while at thicknesses of 4 μm and 8 μm, the SPR shifted to 0.62 THz and 0.55 THz, respectively. Specifically, we observed a frequency shift from 0.69 THz without r-GO to 0.55 THz at a thickness of 8 μm. Also, the THz–TDS measurements without and with r-GO show that the signal amplitude is reduced for the t = 8 μm case compared to the t = 0 condition. This is more likely due to increased attenuation of the THz pulse by the thicker r-GO layer (Fig. 3c), because the addition of r-GO on the array of dipole cavities modifies the effective refractive index by changing its dielectric environment, resulting in a red shift in the resonance condition (0.69 THz to 0.55 THz). These results demonstrate the tunability of the SPR frequency for the different thicknesses of r-GO as a coating on top of the dipole cavities. Therefore, sensors based on arrays of dipole cavities, with their simplicity and compactness, are an attractive option for miniaturized sensing applications. The experimental results presented in this study are in good agreement with the simulation results, except for a slight mismatch in the presence of r-GO. This can be attributed to the fact that r-GO coatings have an inherent porosity, while in the simulations it is treated as a nonporous, homogeneous medium. Also, the small discrepancies with the experimental data can arise from limitations in THz measurements, where information is lost due to the truncation of transmitted THz pulses to avoid influence of etalon pulses during the FFT analysis. Additionally, nonuniformity in device fabrication technology, such as variations in hole geometry, metallization depth, and vertical profile, further contribute to the potential mismatch between simulations and measurements. Nonetheless, our results demonstrate that the proposed dipole cavities structure can be utilized as a versatile platform for sensing the r-GO and modulation applications, with the potential for further optimization through the use of more accurate r-GO modeling.

**Figure 4:**
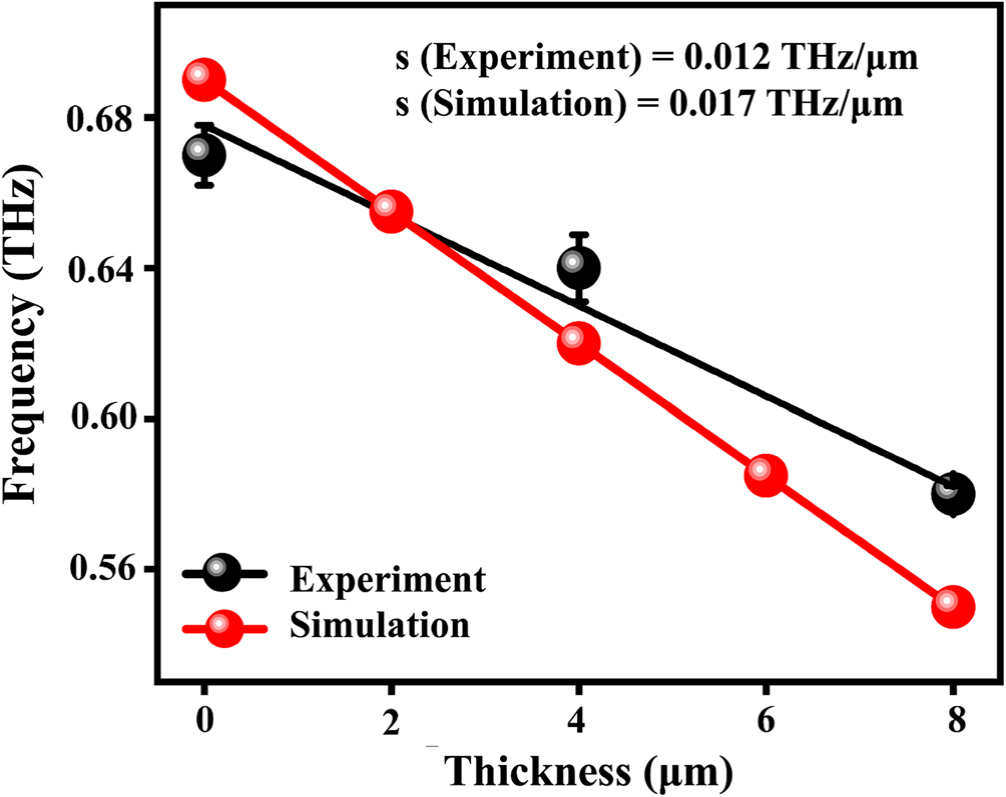
Sensitivity (s) plot for detecting r-GO using dipole cavities. The experimental data (black line) and simulated data (red line) are plotted as frequency shift (THz) versus thickness (µm).

To quantify the SPR frequency shift, we investigated the sensitivity (s) of the dipole cavities in detecting the r-GO film thickness which is shown in Fig. 4. The sensitivity of the dipole cavities is defined as the change in SPR frequency shift per unit thickness of the film, similar to the metasurface based analyte sensing^57^. Both the experimental and simulated data are shown in Fig. 4 using black and red lines respectively. The thickness of the r-GO film on top of the dipole cavities is varied from 0 to 8 µm, and the corresponding SPR frequency shifts are measured. The results show that with an increase in r-GO film thickness, the SPR frequency shift also increases. The sensitivity is determined by analyzing the slope of the plot shown in Fig. 4 which is found to be 0.012 THz/µm (12 GHz/µm) for the experimental data and 0.017 THz/µm (17 GHz/µm) for the simulation (two more data points, 2 µm and 6 µm are considered for the simulation to confirm the trend). The reasonably good agreement between the experimental and simulated results validates the accuracy of the simulation model and reinforces the reliability of the experimental measurements. The higher sensitivity observed in simulations is attributed to the assumed ideal conditions, which do not hold precisely in the experimental setup. There exists a limitation in between simulation and experiment basically due to the inherent porosity and induced electric field distribution of dipole cavities. In simulations a whole slab is considered with constant effective refractive index as extracted from film characterization. However, in case of experiment, the electric field distribution along the vertical axis (for example, z-axis) is not constant, rather decaying away from the air-cavity interface^22^. This means interaction of stronger electric field (nearer to the dipole array) with analyte is actually underestimated in experiment compared to the simulations.

**Figure 5:**
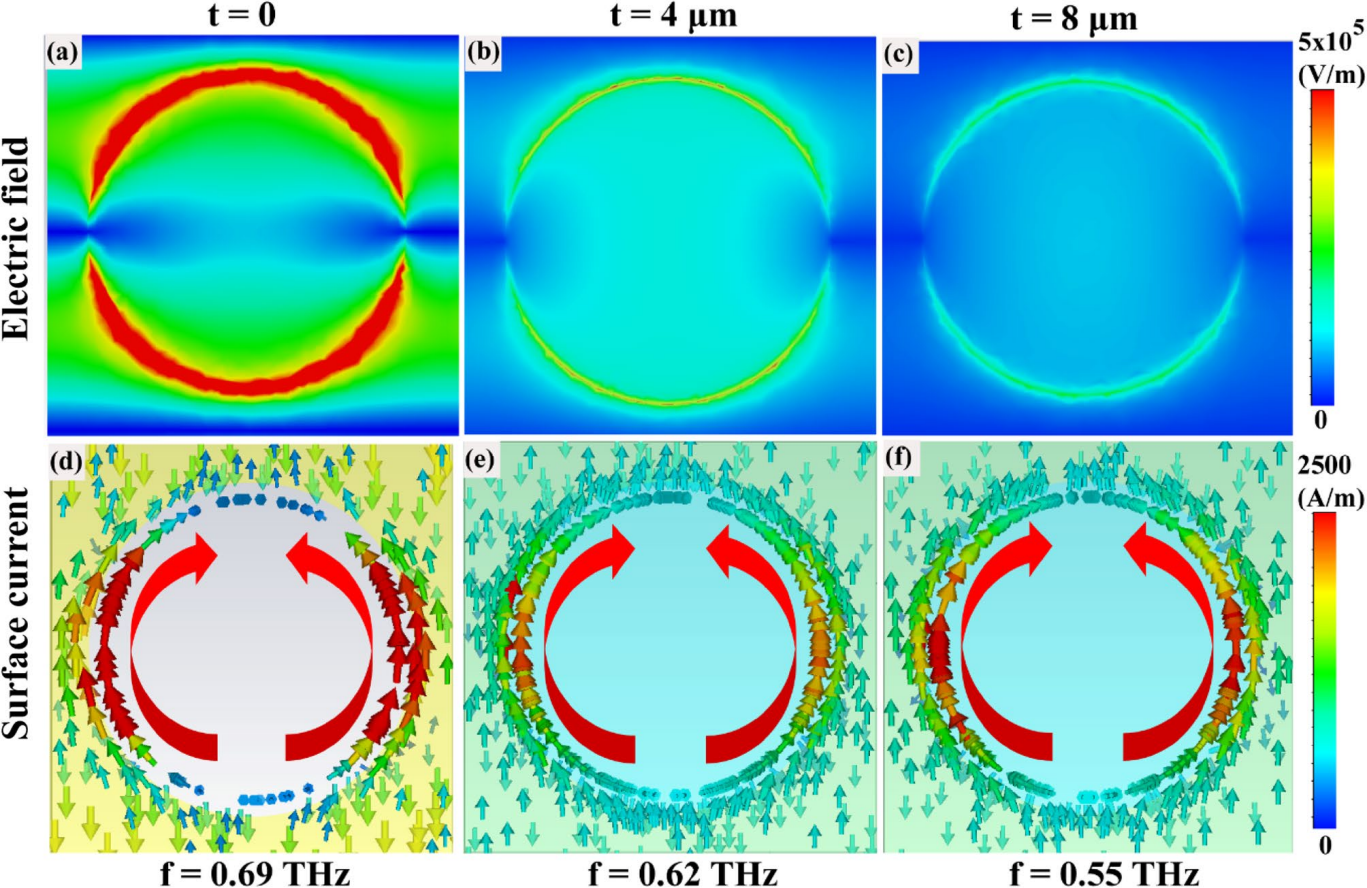
Electric field and Surface current distributions of the array of dipole cavities at different r-GO thickness t = 0 (**a**,**d**), t = 4 μm (**b**,**e**), t = 8 μm (**c**,**f**)) obtained through numerical simulation using CST microwave studio software.

We further analyzed the electric field and surface current distributions at the resonance frequencies to gain a deeper understanding of the interaction between the THz radiation and the r-GO/cavity configurations. This would ultimately affect the SPR frequency shift as shown in Fig. 5. Based on the electric field distribution analysis, it is found that the dipole cavities without r-GO exhibit higher field confinement at the resonance frequency, as shown in Fig. 5a. However, as the thickness of r-GO is increased (t = 4 μm and t = 8 μm), the field confinement is decreased, as illustrated in Fig. 5b,c. The loss in r-GO film affect the field confinements, which further broadens the resonance peak and leads to lesser electric field confinement. The broadening of the peak suggests that the energy is being dissipated through the r-GO layer, reducing the amount of energy that is confined within the array of dipole cavities. From the surface current distribution, it is observed that circular holes exhibit a dipole-like behavior indicating the nature of the induced charge. A dipole current is observed in all three cases as seen from the surface current distribution. However, the dipole cavities without r-GO (Fig. 5d) display a stronger current density compared to those with r-GO (Fig. 5e,f), suggesting stronger induced currents in the absence of r-GO.

## Conclusions

In summary, we ascertain the potential of planar THz dipole cavities as a convenient platform for r-GO (and other 2D materials) sensing applications. First, we demonstrate a successful reduction of GO to r-GO using Hummer’s method and that has been confirmed by several Nano-characterization techniques. The synthesized r-GO particles are drop casted on a silicon substrate to form r-GO films whose optoelectronic properties in the THz frequency domain are extracted using THz-TDS. Further, an array of plasmonic dipole cavities are fabricated and characterized in the THz regime demonstrating distinct SPR modes. We further observed SPR frequency detuning as a result of variations in the thickness of r-GO layer coated on top of the dipole cavities which can form a basis for sensing 2D material at THz domain. The experimental (theoretical) sensitivity of the array of dipole cavities is quantified as 12 GHz/µm (17 GHz/µm). Numerically simulated electric field and surface current distributions substantiate the experimentally observed modulations in SPR characteristics due to the controlled presence of thin r-GO films. These findings suggest that plasmonic dipole cavities offer significant opportunities for sensing r-GO and other Nano porous 2D materials, which eventually can contribute to the development of THz nanophotonic sensors.

## Supplementary Information

Supplementary Information.

## Data Availability

The datasets used in the present study are available from the corresponding author upon a reasonable request.
