# Supplementary material for: Terahertz sensing of reduced graphene oxide nanosheets using sub-wavelength dipole cavities
Source: Sci Rep. 2023 Jul 31;13:12374. doi: 10.1038/s41598-023-39498-4 (PMC10390544; doi:10.1038/s41598-023-39498-4)
Supplement: Supplementary file 1 — Supplementary Information. [file 41598_2023_39498_MOESM1_ESM.docx]

**Supplementary Information**

**Terahertz Sensing of reduced graphene oxide nanosheets using sub-wavelength dipole cavities**

Vaishnavi Sajeev^1^, Shreeya Rane^1^, Debal Ghosh^2^, Nityananda Acharyya^1^, Palash Roy Choudhury^1^, Arnab Mukherjee^2^, Dibakar Roy Chowdhury^1^*

^1^Ecole Centrale School of Engineering (ECSoE), Mahindra University, Hyderabad, 500043, India

^2^ Central Glass and Ceramic Research Institute (CGCRI), Kolkata, 700032, India

E-mail: [dibakar.roychowdhury@mahindrauniversity.edu.in](mailto:dibakar.roychowdhury@mahindrauniversity.edu.in)

**Characterization techniques:**

**1. FTIR measurements of both GO and r-GO SEM and EDAX of GO**

The FTIR spectra of Graphene Oxide (GO) and reduced graphene oxide (r-GO) are measured and analysed to investigate the functional groups present in them. Fig. S1 shows the FTIR spectrum of GO and r-GO, highlighting the characteristic peaks. FTIR analyses of both GO and r-GO reveal distinct differences in peak intensities and positions. In the FTIR spectrum of GO, a broad and intense peak centered at 3380 cm^-1^ is observed, corresponding to the hydroxyl groups (-OH). However, in the spectrum of r-GO, this peak undergoes a reduction in intensity and shifts to approximately 3416 cm^-1 1^ ^2^. The significant reduction in intensity indicates a reduction in the concentration of hydroxyl groups upon the reduction of GO to r-GO. Furthermore, the carboxyl peak observed at 1740 cm^-1^ in the GO spectrum has completely disappeared in the r-GO spectrum. The disappearance of the carboxyl peak confirms the efficient reduction of GO, leading to the elimination of these oxygen-containing functional groups. Apart from that the carbonyl peak at 1620 cm^-1^ is lowered in intensity and the –C-O peak at 850 and 1050 cm^-1^ are also considerably less intense, indicating the removal or transformation of oxygen-containing functional groups.


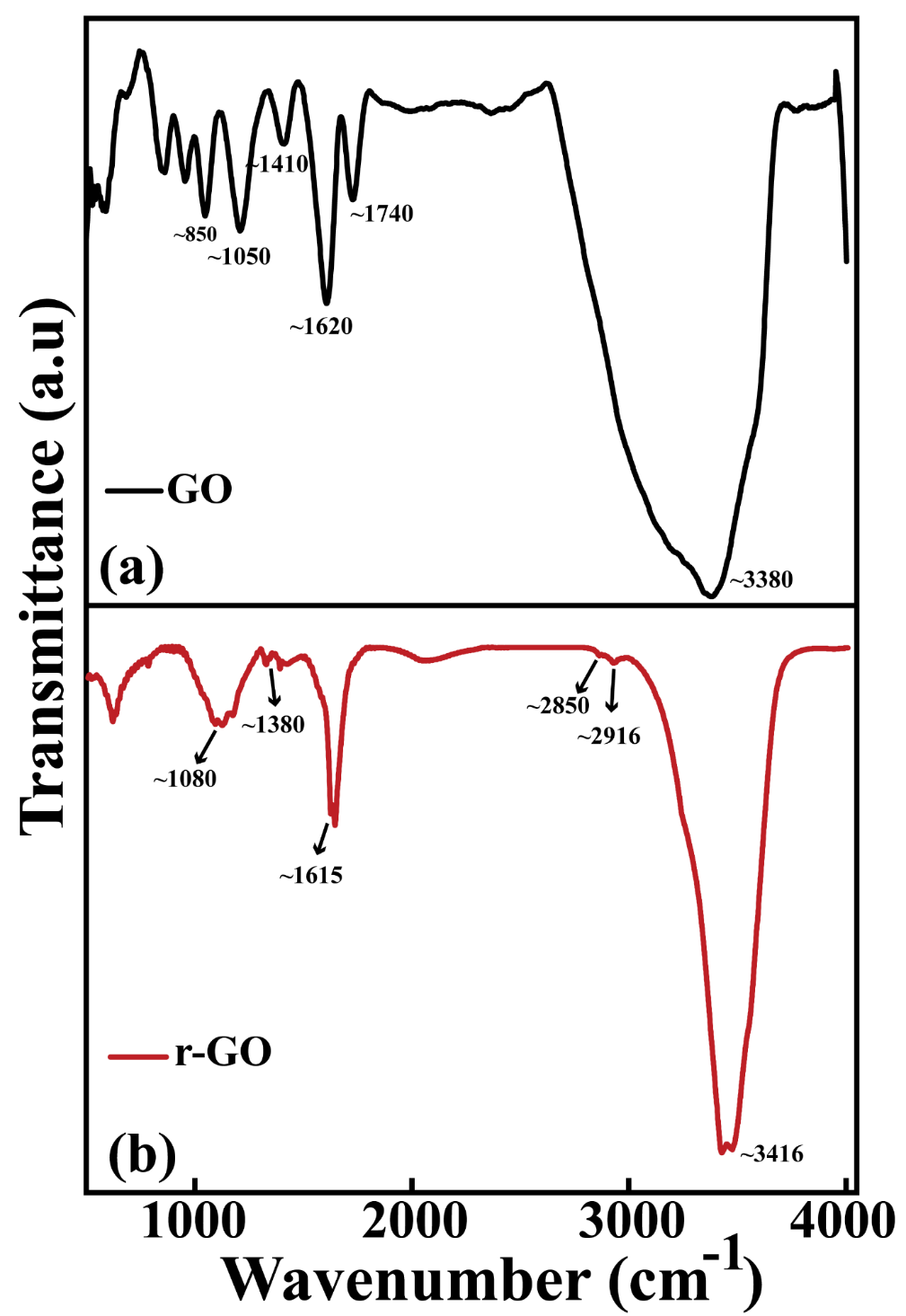


*Fig. S1: FTIR measurements of (a) GO (black) and (b) r-GO (red)*

**2. SEM of GO**

The SEM image of GO shows a sheet-like structure ^3^ and a rough surface along with the presence of wrinkles and folds on the surface as shown in Fig. S2.


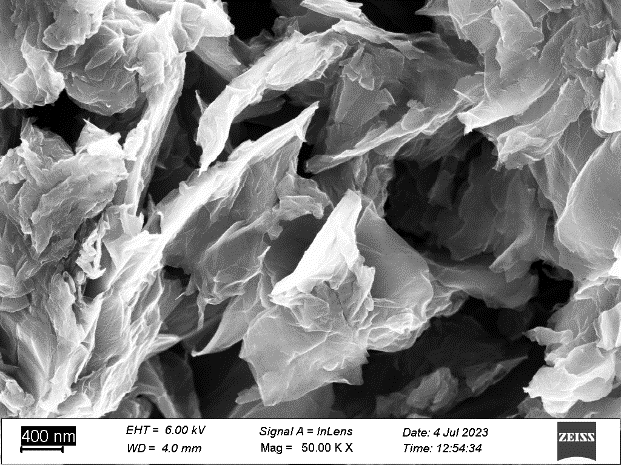

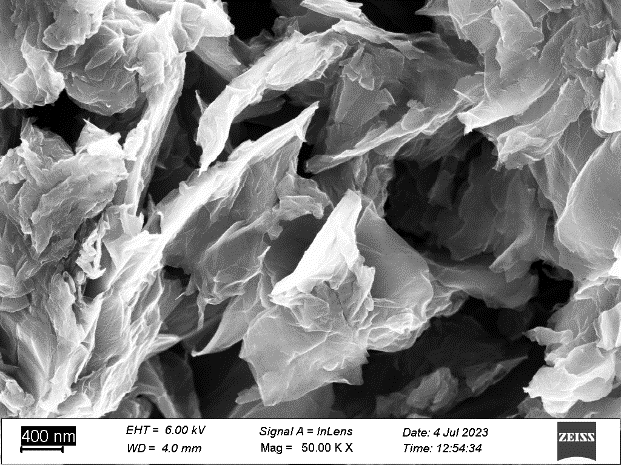


*Fig. S2: SEM image of GO*

**3. EDAX of GO**


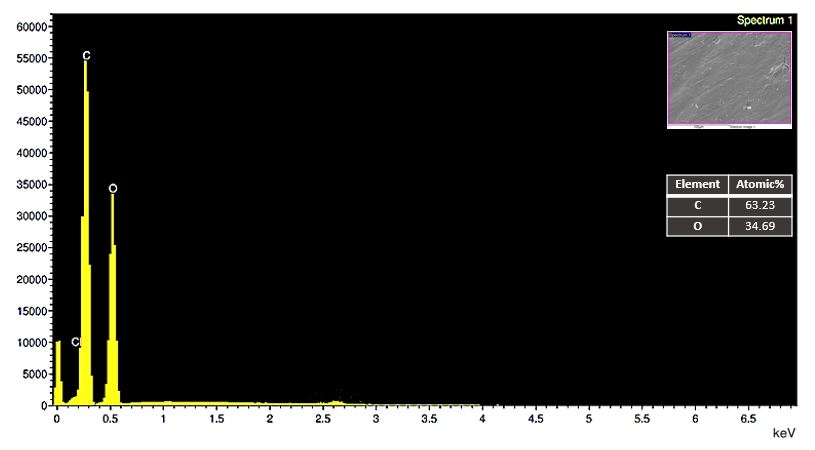


*Fig. S3: EDAX of GO showing the atomic % of carbon and oxygen*

The EDAX analysis of the GO sample indicates that it primarily consists of 63.23% carbon and 34.69% oxygen (atomic percentage) as shown in Fig. S3 ^4^

**References**

1. Andrijanto, E., Shoelarta, S., Subiyanto, G. & Rifki, S. Facile synthesis of graphene from graphite using ascorbic acid as reducing agent. *AIP Conf. Proc.* **1725**, (2016).

2. Emiru, T. F. & Ayele, D. W. Controlled synthesis, characterization and reduction of graphene oxide: A convenient method for large scale production. *Egypt. J. Basic Appl. Sci.* **4**, 74–79 (2017).

3. Chen, Z. *et al.* Modeling shrinkage and creep for concrete with graphene oxide nanosheets. *Materials (Basel).* **12**, (2019).

4. Olumurewa, K. O., Olofinjana, B., Fasakin, O., Eleruja, M. A. & Ajayi, E. O. B. Characterization of High Yield Graphene Oxide Synthesized by Simplified Hummers Method. *Graphene* **06**, 85–98 (2017).

.
